# Contamination in Reference Sequence Databases: Time for Divide-and-Rule Tactics

**DOI:** 10.3389/fmicb.2021.755101

**Published:** 2021-10-22

**Authors:** Valérian Lupo, Mick Van Vlierberghe, Hervé Vanderschuren, Frédéric Kerff, Denis Baurain, Luc Cornet

**Affiliations:** ^1^InBioS-PhytoSYSTEMS, Eukaryotic Phylogenomics, University of Liège, Liège, Belgium; ^2^InBioS, Center for Protein Engineering, University of Liège, Liège, Belgium; ^3^Plant Genetics, TERRA Teaching and Research Center, Gembloux Agro-Bio Tech, University of Liège, Liège, Belgium

**Keywords:** sequencing, assembly, contamination, genomes, databases, NCBI RefSeq, phylogenomics

## Abstract

Contaminating sequences in public genome databases is a pervasive issue with potentially far-reaching consequences. This problem has attracted much attention in the recent literature and many different tools are now available to detect contaminants. Although these methods are based on diverse algorithms that can sometimes produce widely different estimates of the contamination level, the majority of genomic studies rely on a single method of detection, which represents a risk of systematic error. In this work, we used two orthogonal methods to assess the level of contamination among National Center for Biotechnological Information Reference Sequence Database (RefSeq) bacterial genomes. First, we applied the most popular solution, CheckM, which is based on gene markers. We then complemented this approach by a genome-wide method, termed Physeter, which now implements a *k*-folds algorithm to avoid inaccurate detection due to potential contamination of the reference database. We demonstrate that CheckM cannot currently be applied to all available genomes and bacterial groups. While it performed well on the majority of RefSeq genomes, it produced dubious results for 12,326 organisms. Among those, Physeter identified 239 contaminated genomes that had been missed by CheckM. In conclusion, we emphasize the importance of using multiple methods of detection while providing an upgrade of our own detection tool, Physeter, which minimizes incorrect contamination estimates in the context of unavoidably contaminated reference databases.

## Introduction

Genome contamination, defined here as the accidental inclusion of sequences from other organisms or the misclassification of sequences in public repositories, is a problem having attracted much attention in the recent literature (see for instance, Kahlke and Ralph, 2018; Lu and Salzberg, 2018; Breitwieser et al., 2019; Low et al., 2019). Hence, it is notoriously known that contamination of genome-scale datasets can lead to false conclusions, and such cases have been reported in numerous publications (e.g., Laurin-Lemay et al., 2012; Merchant et al., 2014; Koutsovoulos et al., 2016). Nowadays, many algorithms are available to detect contaminants in complete genomes, e.g., Kraken 2 (Wood et al., 2019), CheckM (Parks et al., 2015), Physeter (Cornet et al., 2018), ConFindR (Low et al., 2019), and BASTA (Kahlke and Ralph, 2018). By studying the phenomenon in Cyanobacteria, we have shown that different methods sometimes yield widely different estimates of the contamination level (Cornet et al., 2018). As this result is explained by differences between the respective algorithms or databases, we argued that the use of multiple methods is the best way to detect contaminant sequences (Cornet et al., 2018). In contrast, relying on a single method of detection, even if very well designed and popular, always bears a danger of systematic error, which can eventually lead to the spread of sequences of incorrect taxonomy into public databases. The objective of this Perspective is to highlight the importance of using multiple methods of detection when assessing contamination in genomic studies.

To this end, we investigated the results of the most cited tool (3,532 citations as of September 2021 according to Google Scholar) in the field of contamination detection, CheckM (Parks et al., 2015). The latter is frequently the only method used in genome-scale studies, for example in the Genome Taxonomy Database (GTDB) project, in which specific genomes are selected as type organisms for the community (Parks et al., 2018). We chose to estimate the contamination level of bacterial genomes from the reference sequence database of the National Center for Biotechnological Information (NCBI), Reference Sequence Database (RefSeq; O’Leary et al., 2016; Haft et al., 2018), not only because this resource is frequently used by many researchers (Nasko et al., 2018), but also because it has been reported to be affected by sequence contamination (Cornet et al., 2018; Breitwieser et al., 2019; Pasolli et al., 2019; Zhu et al., 2019). Here, we first evaluated the contamination level of this database using CheckM, and then compared these estimates, for 12,326 results that we considered as potentially dubious, to those obtained with an upgrade of Physeter, a decontamination tool introduced in Cornet et al. (2018).

## CheckM Yields Potentially Dubious Results for 12,326 Genomes in NCBI RefSeq

**FIGURE 1:**
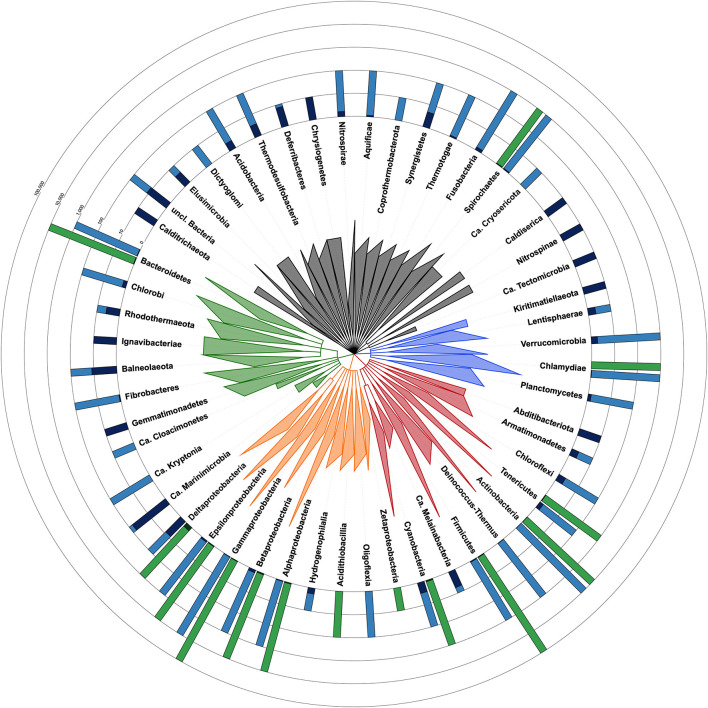
Taxonomic tree of the bacterial domain showing the fraction of contaminated genomes in each phylum with each method. Taxon identifiers of the 111,088 RefSeq bacterial genomes were passed to NCBI Common Tree tools to construct the tree [parameters: (1) include unranked taxa, (2) expand all]. Tree visualization was performed with iTOL and branches were collapsed at the taxonomic levels reported in the tree. Triangles are proportional to taxonomic depth. Proteobacteria are colored in orange, FCB group in green, Terrabacteria in red, PVC group in blue and the other phyla in dark gray. Green barplots are for genomes evaluated with CheckM and blue barplots are for Physeter. The fraction of genomes with a contamination level <5% is shown in a light color whereas those ≥5% are shown in a dark color. The number of genomes evaluated with each method is indicated by the height of the barplot on a ceiled logarithmic scale. For simplicity, the estimates for Ca. Saccharibacteria (2 contaminated and 12 uncontaminated genomes), candidate division NC10 (2 contaminated genomes), Ca. Atribacteria (2 contaminated genomes), and Ca. Bipolaricaulota (1 contaminated genome) are included in unclassified Bacteria. Completely contaminated phyla (e.g., Caldiserica, Nitrospinae, and Kiritimatiellaeota) are generally represented by very few genomes (i.e., one to three genomes). Among the more extensively studied phyla (11 to 37,487 genomes), some appear to be extremely contaminated, such as Balneolaeota, Synergistetes, and Chloroflexi, with, respectively, 54.5, 33.3, 16.9% of contaminated genomes, whereas other phyla are characterized by a very low contamination level, including Cyanobacteria (2.8%), Gammaproteobacteria (0.6%), or Chlamydiae (0.3%).

CheckM estimates the contamination level in a given genome by counting duplications of single-copy and taxon-specific gene markers (Parks et al., 2015). This requires a phylogenetic placement of the genome, based on ribosomal protein genes, in order to determine its taxon and derive the appropriate marker set (Parks et al., 2015). However, for 12,326 bacterial genomes among the 111,088 of RefSeq (Haft et al., 2018), this first step of the algorithm yields a dubious taxon, which has the potential to affect the contamination estimate. In detail, CheckM results were considered dubious for at least one, frequently several, of the four following reasons (Supplementary Table 1: https://doi.org/10.6084/m9.figshare.13139810): (1) the CheckM taxon obtained by phylogenetic placement is ambiguous when compared to the NCBI taxon, even if closely related (e.g., same phylum; 9,257 cases), (2) the CheckM taxon is of a too high level (e.g., “bacteria”) to be useful in practice (2,967 cases), (3) the CheckM taxon is “incorrect” (e.g., different phylum) with respect to the NCBI taxon or both taxa are uninformative (77 cases), and (4) the estimated contamination level is ≥20% (25 cases), which is the upper tested limit of detection for CheckM (per documentation). In the latter case, CheckM results can be erroneous because its phylogenetic placement is affected by an array of supernumerous ribosomal genes belonging to the contaminants. Owing to these reasons, the current release of CheckM produces reliable estimates for only 14 phyla whereas these are questionable for 38 phyla (Figure 1). However, the accuracy of CheckM on the remaining 98,801 genomes of RefSeq has not been investigated here.

## Physeter as a Second Estimator of the Contamination Level

**FIGURE 2:**
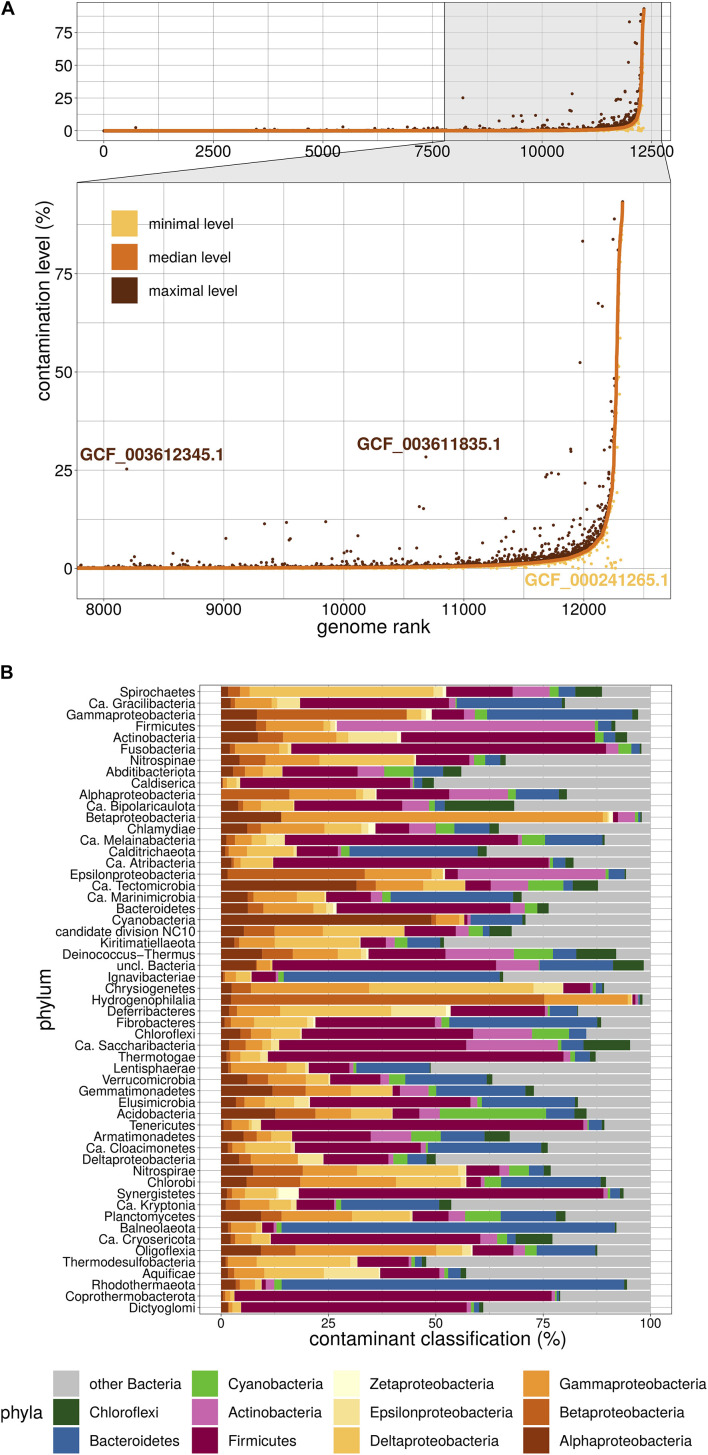
Overview of Physeter properties. **(A)** Distribution of contamination levels assessed by Physeter in *k*-fold mode. Genomes are ranked from the lowest to highest median level of contamination. Median levels are shown in a solid orange line, while minimal and maximal levels are represented as yellow and brown dots, respectively. GCF_003612345.1 and GCF_003611835.1 are examples of genomes having a low median level of contamination with some independent estimations showing a higher contamination level. The opposite case is illustrated with GCF_000241265.1. **(B)** Taxonomic distribution of contaminating sequences within each phylum. The relative contributions of each contaminating phylum were first averaged by genome over all 10 *k*-folds, then these genome-wise averaged values were averaged by tested phylum over all genomes.

We then used Physeter to estimate the contamination level of the 12,326 dubious genomes. Physeter features a MEGAN-like (Huson et al., 2007) Last Common Ancestor (LCA) algorithm that uses DIAMOND blastx (Buchfink et al., 2015) results to compute its estimates. Here, we upgraded its heuristics to overcome the unavoidable presence of contaminated genomes in reference databases. In practice, a sliding window splits the reference database into 10 partitions, and Physeter returns the median contamination level of 10 independent estimations, each one based on 90% of the database. This *k*-fold approach allowed us to identify false positive results only driven by a few contaminated genomes in the reference database (Figure 2A). For instance, the assemblies GCF_003612345.1 and GCF_003611835.1 have a low median level of contamination, even if some independent estimations (Figure 2A) show a higher level. The opposite is also observed (Figure 2A), with some contaminated genomes leading to false negative results (see Supplementary Additional File 1). Overall, Physeter minimizes the estimation biases due to overlooked contamination while maintaining the diversity of the reference database (Supplementary Figure 1).

## Taxonomic Errors and Rare Genomes

According to Physeter, 107 RefSeq genomes (among the 12,326) presented very low levels of the organism expected from the associated NCBI taxon. First, these “taxonomic errors” may correspond to genomes that are misclassified by the NCBI (e.g., GCF_900453015.1). Such misclassifications should also be considered as contamination because misclassified genomes are susceptible to be incorporated in downstream studies under a wrong taxonomy, which could be very damaging to biological conclusions (Laurin-Lemay et al., 2012). Second, taxonomic errors can also stem from genomes that are so contaminated that the sequences of the expected organism are overwhelmed by the foreign sequences (e.g., GCF_003264215.1). Third, some genomes belong to a taxon that is so rare in genome databases that they only match themselves, which is not allowed by the Physeter algorithm and thus leads to low levels of the expected organism (e.g., GCF_000226295.1), including 45 genomes tagged as “unclassified Bacteria” by the NCBI. In practice, distinguishing between the three cases is very difficult. Among the 107 genomes, 65 were left unclassified by CheckM (i.e., identified as “bacteria” or “root”) with a low level of contamination (median 1.1%), whereas Physeter found high contamination levels (median 14.6%) for these 65 cases. To deal with those 107 problematic genomes, we re-ran Physeter using the GTDB taxonomy (Parks et al., 2018) as an alternative and let the tool determine the main organism itself, just like CheckM usually does (see Supplementary Table 1). In theory, the use of GTDB should help us to discriminate between taxonomic errors and rare genomes, though in practice it does not. This is so because 76 genomes (among the 107) are representative genomes in GTDB, which have been decontaminated based on CheckM results alone. On the other hand, Physeter’s auto-detection mode is not compatible with its self-match skipping feature. Therefore we cannot make a decision on these 107 complex cases. The take-home message of this section is that estimating the contamination level in the case of rare genomes or taxonomic errors is very difficult, especially when interconnected tools are used.

## The Case for Corroborated Estimates

Based on the recommendations established by the Genomic Standards Consortium (Bowers et al., 2017), we used a threshold of 5% to decide if a genome is contaminated. CheckM and Physeter results can only be compared in the context of this specific cutoff, since the two algorithms are very different and hardly comparable in terms of contaminant percentage. Moreover, while CheckM is based on taxon-specific marker sets, Physeter probes the whole genomes. Nevertheless, the results can be divided into four categories based on the maximum contamination threshold of 5%: (1) both methods identify <5% of contaminants (11,759 genomes), (2) CheckM alone identifies ≥5% of contaminants (384 genomes), (3) Physeter alone identifies ≥5% of contaminants (133 genomes), and (4) both methods identify ≥5% of contaminants (46 genomes). The two methods are thus in agreement for 95.77% of the 12,326 dubious genomes. The discrepancies were expected based on our previous results on Cyanobacteria, where we compared six different detection methods (Cornet et al., 2018). Even if numerically minor, they confirm the importance of using multiple methods of detection when estimating contamination levels. Schematically, the intersection of the methods (i.e., corroboration) increases the certainty that a given genome is contaminated, hence reducing false positives, whereas the union maximizes the power of detection, hence reducing false negatives. The choice of the intersection or of the union is dependent on the goal of study, as both options have their drawbacks, either more false negatives or more false positives, respectively. At this stage, it is difficult to decide “which method is right” between CheckM and Physeter. One way would be to perform a metagenomic binning on the genomes for which they disagree. However, sequencing reads are not publicly available for more than half of these genomes (only 41.3 and 45.1% for category 2 and 3, respectively), and these genomes being lowly contaminated, the foreign bins are too small to be accurately classified by any tool.

Physeter presents the advantage of labeling the individual sequences and thus offers the possibility to explore the taxonomy of the contaminants. These are very diversified, with a median of 45 different contaminant phyla per phylum (over the 10 *k*-fold replicates). Firmicutes appear to be the major contaminant of various phyla (Figure 2B), such as Tenericutes (75.1% of the contaminant sequences), Fusobacteria (73.3%), Synergistetes (70.9%), or Thermotogae (68.9%). Reciprocally, the major contaminant of Firmicutes genomes are Actinobacteria (60.1%). Biological traits like sheath thickness or the abundance of co-living organisms can explain the nature of the contaminants and the fact that some taxa have a higher propensity for contamination, the latter being also affected by uneven sampling of lifestyles in RefSeq (e.g., lots of clinical samples).

## Discussion

In this study, we have only looked at bacterial genomes contaminated by other bacterial sequences. However, the situation can be more complex, for instance in metagenomic samples including small eukaryotes where contaminations can remain unnoticed by most algorithms to the exception of Kraken (Wood et al., 2019), BlobToolKit (Challis et al., 2020), a workflow developed for eukaryotes, and Physeter (Cornet et al., 2018). As a case in point, we provide a protocol to construct a database containing representative genomes from the three domains of life and study contamination in complex samples with Physeter (see Supplementary Additional File 2). Based on the results of the present study, even the most curated database publicly available, RefSeq, includes 1,395 significantly (≥5%) contaminated genomes (considering the union of CheckM and Physeter results), which translates to 1.25% of the genomes. This low percentage should not be considered as a comforting result because even a single contaminated genome can lead to false interpretations (Bemm et al., 2016). Perhaps more critical, since nearly all contamination detection tools use databases derived from public repositories as references [RefSeq (Haft et al., 2018) for Kraken (Wood et al., 2019), Integrated Microbial Genomes (IMG; Markowitz et al., 2012) for CheckM (Parks et al., 2015), Ensembl (Hubbard et al., 2002) for the first version of Physeter (Cornet et al., 2018), RefSeq (Haft et al., 2018) for ConFindR (Low et al., 2019), RefSeq (Haft et al., 2018) for BASTA (Kahlke and Ralph, 2018)], the reliability of the detection hinges on the quality of these public databases. To our knowledge, Physeter is the only software able to robustly detect contaminations at a genome-wide scale when using a moderately contaminated database as a reference.

Considering the low level of contaminated genomes in RefSeq, one could conclude that the risk to include contaminants in a study, due to reliance on a single method of detection, is also low. Nevertheless, researchers are by essence more interested in particularities than by generalities, and even small amounts of contaminants have the potential to lead to exciting but false conclusions. That is why we argue that a “second opinion” should be considered when searching for contaminating sequences, especially as long as genome reference databases are not completely devoid of contamination (Pasolli et al., 2019; Zhu et al., 2019).

## Methods

111,088 genomes were downloaded from RefSeq on the 9th of March 2019, regardless of their sequencing status. These genomes were analyzed with CheckM using the typical automatic workflow option lineag_wf. CheckM automatically places the queried genomes in a reference tree through the concatenation of predicted ribosomal proteins. The completeness and contamination levels are then estimated by searching for lineage specific marker genes provided with the software. CheckM uses 5,656 genomes from a decontaminated version of IMG dating from 2015 (Parks et al., 2015).

For Physeter analyses, we first built a DIAMOND blastx database corresponding to the 177,288 genomes of the Kraken2 database (Wood et al., 2019; Supplementary Table 2: https://doi.org/10.6084/m9.figshare.13139819). This very comprehensive database is composed for a large part of RefSeq genomes, after curation by the authors (Wood et al., 2019). Yet it only includes bacterial genomes, which prevents us from analyzing archaeal genomes here. Moreover, CheckM indicated that 685 genomes of this database are contaminated, which motivated our choice of a leave-one-out approach. The queried genomes were then split into pseudo-reads of 250 nt, BLASTed against the protein database, and labeled by computing the LCA of each pseudo-read based on its best hits (excluding self-matches), provided that they yielded a bit-score ≥ 80 and within 95% of the bit-score of the first hit (MEGAN-like algorithm; Huson et al., 2007). As in Cornet et al. (2018), we chose to set the minimal number of best hits to 1 for computing LCAs. For the 107 misclassified genomes on the NCBI, we ran Physeter using a local mirror of the GTDB taxonomy (Parks et al., 2018; release 202) instead of the NCBI Taxonomy. Taxa were attributed through the “auto-detect” option and the “labeller” was constructed using all available GTDB phyla, except for Proteobacteria, which were split into their constituting classes instead.

## Data Availability Statement

Publicly available datasets were analyzed in this study. This data can be found here: https://doi.org/10.6084/m9.figshare.13139810.v2; https://doi.org/10.6084/m9.figshare.13139819.v1; https://metacpan.org/dist/Bio-MUST-Apps-Physeter.

## Author Contributions

LC and DB conceived the study. LC, VL, MV, and DB developed Physeter. VL performed all analyses and drew the figures. LC supervised the study. LC, VL, and DB wrote the manuscript. All authors read and approved the final manuscript.

## Conflict of Interest

The authors declare that the research was conducted in the absence of any commercial or financial relationships that could be construed as a potential conflict of interest.

## Publisher’s Note

All claims expressed in this article are solely those of the authors and do not necessarily represent those of their affiliated organizations, or those of the publisher, the editors and the reviewers. Any product that may be evaluated in this article, or claim that may be made by its manufacturer, is not guaranteed or endorsed by the publisher.

## Abbreviations

RefSeq: Reference Sequence Database
LCA: Last Common Ancestor
IMG: Integrated Microbial Genome
NCBI: National Center for Biotechnological Information
GTDB: Genome Taxonomy Database.
