# Supplementary material for: Contamination in Reference Sequence Databases: Time for Divide-and-Rule Tactics
Source: Front Microbiol. 2021 Oct 22;12:755101. doi: 10.3389/fmicb.2021.755101 (PMC8570097; doi:10.3389/fmicb.2021.755101)
Supplement: Supplementary file 1 [file Data_Sheet_1.pdf]

Additional file 1: Technical explanation of Physeter algorithm

Background

Physeter is a command-line tool that uses DIAMOND blastx (Buchfink et al., 2015) reports to assess the level of contamination of a genome assembly (see manual). To ensure maximum sensitivity, it is advised to split the genome to analyze into pseudo-reads of 250-250 nt (Cornet et al., 2018). Like BASTA (Kahlke & Ralph, 2018), it is based on a Last Common Ancestor (LCA) algorithm to assign a taxonomy to each pseudo-read of the genome. Its algorithm consists in accumulating the hits for each query, (2) assigning a lineage to these hits (based on NCBI Taxonomy), (3) computing a LCA that is then used to taxonomically annotate the pseudo-read and (4) classifying the pseudo-read to compute the ratio of contaminated pseudo-reads in the genome. Our LCA algorithm was also inspired by MEGAN (Huson et al., 2007) for hit accumulation, using a bit score threshold expressed as a percentage of the highest bit score of the current pseudo-read.

Algorithm description

Classic mode

The first step of the Physeter algorithm is to parse the DIAMOND blastx report (where queries are pseudo-reads). For each query, Physeter evaluates whether the first hit passes all the specified thresholds (i.e., length, percent of identity, bit score). If so, it starts accumulating hits according to the --tax-min-hits and --tax-max-hits (see manual) thresholds, i.e., minimum and maximum numbers of hits to accumulate in order to compute the LCA. If --tax-min-hits and --tax-max-hits are both set to 1, then Physeter only uses the best hit to assign taxonomy (BEST HIT MODE). The highest bit score among the hits is used to initialize the bit score threshold. This bit score threshold itself is computed by multiplying the highest bit score by the --tax-score-mul (MEGAN-LIKE MODE). During hit accumulation, if the genome has a NCBI GCA/GCF accession (see manual), the hits corresponding to the organism are ignored. In contrast, this is not possible when using either the --exp-tax or --auto-

detect option (designed for custom genome assemblies; see below). Hit accumulation can stop for different reasons: 1) minimum of hit is not reached, thus no LCA is computed and Physeter goes to the next query, 2) maximum of hit is reached, if minimum of hit is reached too, therefor LCA is computed, if not, no LCA is computed, 3) the bit score of a hit is lower than the bit score threshold and like point 2) LCA is compute or not either if minimum of hit is reached or not. Then, Physeter uses a local mirror of the NCBI Taxonomy to fetch the lineages of all accumulated hits in order to compute the LCA. The optional --tax-min-lca-freq threshold can be applied to discard minor lineages incongruent with those encountered in majority. This threshold works at any taxonomic level, which makes it very efficient at determining the most precise LCA. For diagnostic purposes, Physeter keeps track of the LCA assigned to each query (or lack of) and the number of hits used in the taxonomical computation.

The second step is to determine if the assigned taxonomy (LCAs) of the pseudo-reads corresponds or not to the organism taxonomy. The organism taxonomy can be determined in three ways: 1) based on its NCBI GCA/GCF accession in the case of public genome assemblies, 2) user-specified using the --exp-tax option for custom genomes for which one approximately knows the taxonomy, 3) through auto-detection (--auto-detect option) based on the most abundant LCA identified during the first step. In contrast to CheckM (Parks et al., 2015), which uses ribosomal phylogenetic placement followed by the detection of clade-specific sets of about hundreds marker genes to evaluate its contamination level, Physeter considers the entire set of pseudo-reads of the genome under analysis. To this end, the organism taxonomy and the pseudo-read LCAs are remapped at a higher taxonomic level, using a taxonomic labeller defined as a list of high-ranking NCBI taxa. For some ambiguous taxa with the same label at different taxonomic levels (e.g., Actinobacteria), the --greedy-taxa option can be used to decide which level to use when remapping pseudo-reads (see manual). After labelling, pseudo-reads are classified into one of four categories: 1) ‘self’ if the labels are identical between the organism and the pseudo-read, 2) ‘contaminated’ if the labels are different, 3) ‘unknown’ if no label could be assigned to the pseudo-read (e.g., if the LCA is too high-ranking, such as ‘cellular organisms’, 4) ‘unclassified’ if no LCA could be computed due to a lack of hits to the reference DIAMOND database. Interestingly,

the sensitivity of Physeter is not affected by the number of hits used to compute LCAs. Indeed, while the unclassified fraction increases with the number of hits, some groups with very high fractions of classified sequences are also among those with the highest numbers of hits (Fig. S1, e.g., Firmicutes). The eight taxonomic groups with no genome fraction identified as “self” (Fig. S1, e.g., Nitrospinae) are rare phyla represented by a maximum of two genomes (five with one and three with two genomes). Since our approach does not take into account self hits, zero to one reference genomes are available for classification of these organisms, which leads to the observed lack of “self”. As expected, the abundance of genomes from a given phylum positively influences the number of hits, but only moderately. Hence, even if highly represented phyla attract more hits, some less represented phyla (e.g., Spirochaetes) are also characterized by high numbers of hits (Fig. S1).

k-fold mode

The *k*-fold mode allows users to systematically ignore subsets of the DIAMOND database, so as to identify the reference genomes leading to false detection. The list of NCBI GCA/GCF accessions used to construct the database is passed to Physeter using --kfold option. Then, accessions are shuffled and split into 10 equal-sized subsets. The functioning of the algorithm described in *Classic mode* stays the same except that Physeter runs 10 times and, for each run, hits that belong to the subset to be ignored are skipped during hit accumulation. Finally, non-parametric statistics are computed for each sequence category.

Dataset

The DIAMOND reference database used in this study is based on the Kraken2 database (Wood et al., 2019) and contains 177,288 genomes. The 111,907 tested genomes were downloaded from RefSeq on the 9th of March 2019.

To download and run Physeter, see https://metacpan.org/dist/Bio-MUST-Apps-Physeter.

References

Buchfink, B., Xie, C., & Huson, D. H. (2015). Fast and sensitive protein alignment using DIAMOND. *Nature Methods*, *12*(1), 59–60. https://doi.org/10.1038/nmeth.3176

Cornet, L., Meunier, L., Vlierberghe, M. V., Léonard, R. R., Durieu, B., Lara, Y., Misztak, A., Sirjacobs, D., Javaux, E. J., Philippe, H., Wilmotte, A., & Baurain, D. (2018). Consensus assessment of the contamination level of publicly available cyanobacterial genomes. *PLOS ONE*, *13*(7), e0200323. https://doi.org/10.1371/journal.pone.0200323

Huson, D. H., Auch, A. F., Qi, J., & Schuster, S. C. (2007). MEGAN analysis of metagenomic data. *Genome Research*, *17*(3), 377–386. https://doi.org/10.1101/gr.5969107

Kahlke, T., & Ralph, P. J. (2018). BASTA – Taxonomic classification of sequences and sequence bins using last common ancestor estimations. *Methods in Ecology and Evolution*, *10*(1), 100–103. https://doi.org/10.1111/2041-210X.13095

Parks, D. H., Imelfort, M., Skennerton, C. T., Hugenholtz, P., & Tyson, G. W. (2015). CheckM: Assessing the quality of microbial genomes recovered from isolates, single cells, and metagenomes. *Genome Research*, *25*(7), 1043–1055. https://doi.org/10.1101/gr.186072.114

Wood, D. E., Lu, J., & Langmead, B. (2019). Improved metagenomic analysis with Kraken 2. *BioRxiv*, 762302. https://doi.org/10.1101/762302

Supplementary Figures

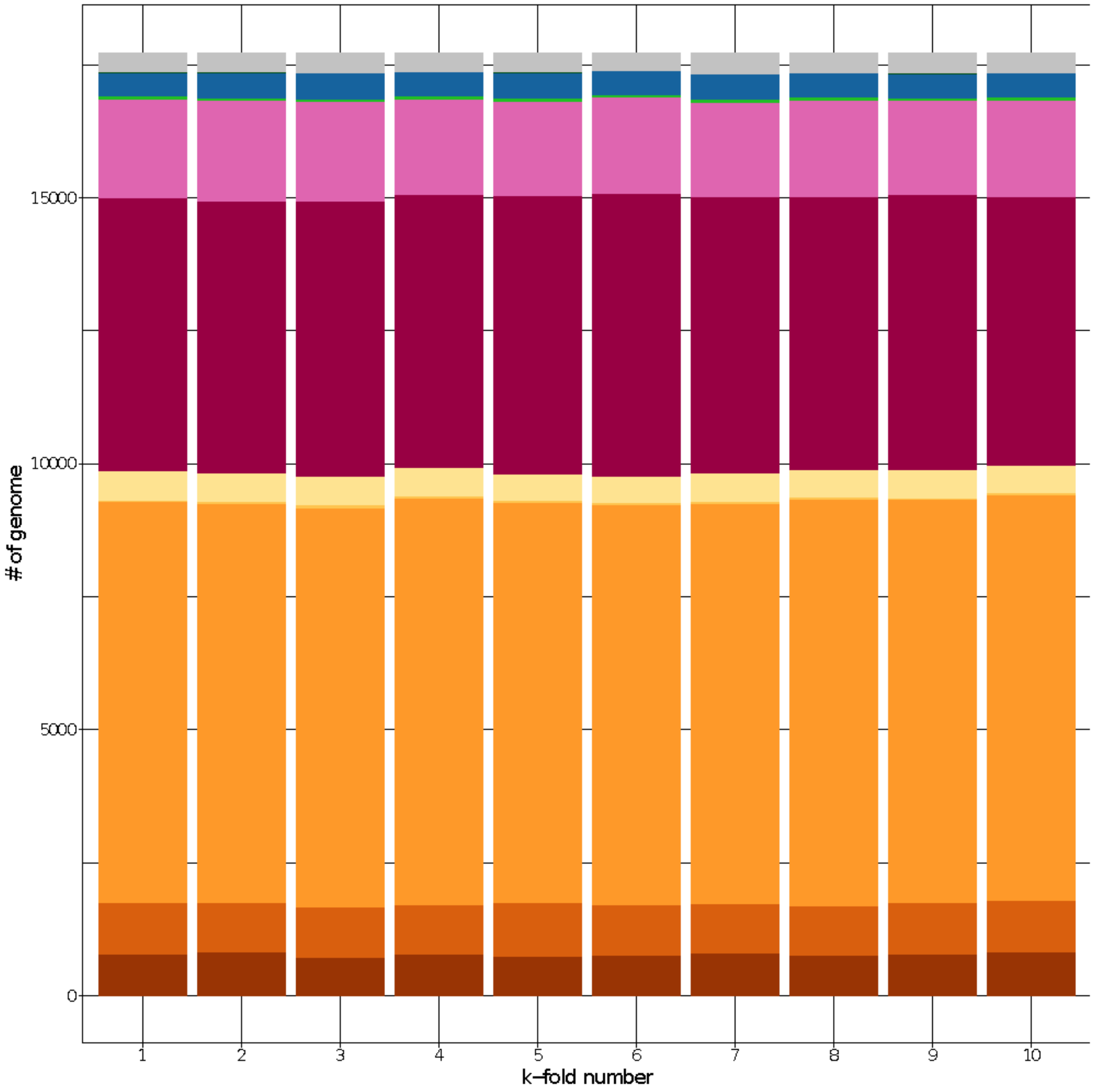

Fig S1. **Taxonomic diversity of 10 equal-sized partitions randomly generated by Physeter in an examplative *k*-fold analysis**. Each of these subsets is left out in turn so as to estimate the sensitivity of the results towards database composition.

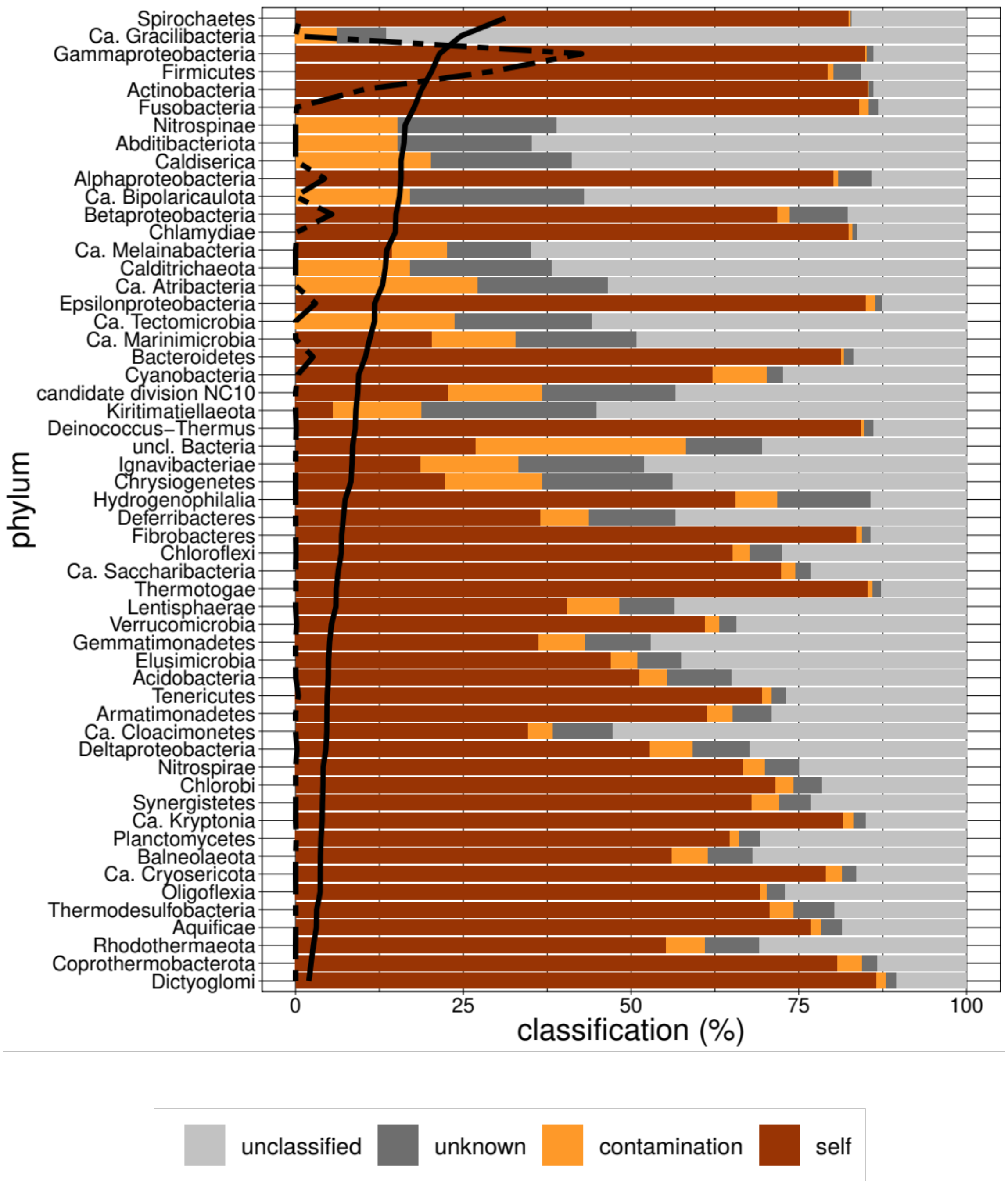

Fig S2. **Relative distribution of the four classification categories for each phylum**. Percentages are the average of the respective median values computed in *k*-fold mode for each genome within the phylum. The average median value of the average number of hits used to compute a LCA is represented with a solid black line, which is further used to rank the phyla. The dashed black line shows the fraction of genomes represented by each phylum in the database. Such a fraction computed on cumulative lengths (in amino acids) rather than numbers of genomes (counts) would have yielded a very similar curve.

Additional file 2: Running Physeter on complex samples

Installation

For installation and dependencies, see manual at: https://metacpan.org/dist/Bio-MUST-Apps-Physeter.

Input files

Install a local mirror of the *NCBI Taxonomy* or the *GTDB Taxonomy*.

```
$ setup-taxdir.pl --taxdir=ncbi-taxdump/
$ setup-taxdir.pl --taxdir=gtdb-taxdump/ --source=gtdb
```

Building the DIAMOND database

Get prokaryote proteome download links at:
https://figshare.com/articles/dataset/Datasets_for_L_onard_et_al_ToRQuEMaDA_Tool_for_Retrieving_Queried_Eubacteria_Metadata_and_Dereplicating_Assemblies/13238936/2.

Decompress the prokaryote archive file.

```
$ tar -xf tqmd_datasets.tar.gz
```

Download and decompress bacterial and archaeal proteomes.

```
$ for f in `cut -f4
  tqmd_datasets/tables/bacteria-151-tax-links.tsv \
  tqmd_datasets/tables/archaea-86-tax-links.tsv`; do wget \
  ${f}/*protein.faa.gz ; done
$ gunzip *.faa.gz
```

Rename sequence identifiers.

```
$ ls *.faa | perl -nle '($gcf) = m/(GC[AF]\_\d{9}\.\d{1})/ ;
  print "$_\t$gcf"' > file.idm
$ inst-abbr-ids.pl --id-prefix-mapper=file.idm \
  --id-regex=:DEF *.faa
```

Concatenate prokaryote files.

```
$ cat *-abbr.faa > prokaryote.faa
$ rm -f GCF*.faa
```

Download eukaryote proteomes at: https://doi.org/10.6084/m9.figshare.13573424.

Decompress eukaryote proteome files.

```
$ tar -xf Data_set_2.tar.gz
```

Rename sequence identifiers.

```
$ cd Data_set_2/
$ perl -i.bak -nle 's/>.*_(\d+)@(.*)$/>\1|\2/; print' *.faa
```

Concatenate prokaryote and eukaryote files.

```
$ cat Data_set_2/*.faa prokaryote.faa > database.faa
```

Build the `DIAMOND` database.

```
$ diamond makedb --in database.faa -d database
```

Running DIAMOND BLASTX

Before running `DIAMOND`, you have to transform the prokaryotic genome files you want to assess into pseudo-read `FASTA` files. Use `inst-split-fas.pl` from the Bio::MUST::Core distribution to do so. In the example below, the genome will be split into 250-base long pseudo-read sequences without overlap. If your genome has a NCBI GCA/GCF accession, name your `outfile` `assembly_accession.fasta` (e.g., `GCF_000006605.1.fasta`).

```
$ inst-split-seq.pl genome.fasta --out=-split
```

Then run `DIAMOND` as follows. Like the `FASTA` file, name your `BLASTX` report as `assembly_accession.blastx` (e.g., `GCF_000006605.1.blastx`). If your genome file does not have a NCBI GCA/GCF accession, both the `FASTA` file and the `BLASTX` report must have the same basename. The `-f tab` option of DIAMOND will generate a tab-separated file corresponding to the `-outfmt 6` of regular *NBCI-BLAST+*. You can adapt the `-p 10` option (number of CPU threads) to suit your system.

```
$ diamond blastx -d database -q split-genome.fasta -o \
  split-genome.blastx -t ./temp -k 50 -e 1e-10 -f tab -p 10
```

Taxonomic labeller

A taxonomic labeler is used by `physeter.pl` to determine at which taxonomic level you consider a pseudo-read sequence as a contaminant. Note that you have to adjust your labeler depending on the used taxonomy. See examples below:

```
$ head phylum-taxa.idl

unclassified Bacteria
unclassified Archaea
Abditibacteriota
```

```
Acidithiobacillia
Acidobacteria
Actinobacteria
Alphaproteobacteria
Aquificae
Armatimonadetes
Bacteroidetes
```

Command-line options of physeter.pl

Classic mode

Once all input files are correctly prepared, you can simply run `physeter.pl` like this:

```
$ physeter.pl *.blastx --outfile=contam.report \
  --taxdir=ncbi-taxdump/ --taxon-list=phylum-taxa.idl
```

Or using GTDB taxonomy.

```
$ physeter.pl *.blastx --outfile=contam.report \
  --taxdir=gtdb-taxdump/ --taxon-list=phylum-taxa.idl
```

The standard output file of `physeter.pl` is a tab-separated file containing the following sections: (1) organism accession or file name, (2) assigned taxon, (3) % self sequences, (4) % contaminated sequences, (5) % unknown taxon sequences, (6) % unclassified sequences, (7) detail of contaminants, (8) mean number of hits used to classify the pseudo-read sequences.

In addition to the Physeter output file, you can generate for each assayed genome a Kraken-like file, an Anvio-like file, a Krona-compatible file or a LCA (Last Common Ancestor) file, the latter providing the taxonomic affiliation of each pseudo-read.

```
$ physeter.pl *.blastx --outfile=contam.report \
  --taxdir=gtdb-taxdump/ --taxon-list=phylum-taxa.idl \
  --kraken --anvio --krona --lca
```

When your pseudo-read FASTA files are not in the working directory, you can specify their localization using the `--fasta-dir` option.

```
$ physeter.pl *.blastx --outfile=contam.report \
  --fasta-dir=split-fasta/ --taxdir=gtdb-taxdump/ \
  --taxon-list=phylum-taxa.idl
```

If your organism does not have a NCBI GCA/GCF accession but you know approximately its taxonomy, you can specify it with the `--exp-tax` option. Note that the specified taxon must be listed in the file provided through the `--taxon-list` option.

```
$ physeter.pl organism.blastx --exp-tax=Firmicutes \
  --outfile=contam.report --taxdir=gtdb-taxdump/ \
  --taxon-list=phylum-taxa.idl
```

Otherwise, use the `--auto-detect` option.

```
$ physeter.pl organism.blastx --auto-detect \
  --outfile=contam.report --taxdir=gtdb-taxdump/ \
  --taxon-list=phylum-taxa.idl
```

In the basic configuration, `physeter.pl` will assess the contamination status of a pseudo-read sequence using only 1 hit (i.e., *best-hit mode*). If you want to use more than 1 hit (i.e., *MEGAN-like mode*), you can use the `--tax-min-hits` and `--tax-max-hits` options. In the *MEGAN-like mode*, a LCA will be inferred for each pseudo-read sequence.

```
$ physeter.pl *.blastx --outfile=contam.report \
  --taxdir=gtdb-taxdump/ --taxon-list=phylum-taxa.idl \
  --tax-min-hits=2 --tax-max-hits=50
```

You can use `--tax-score-mul` and `--tax-min-lca-freq` options to fine tune LCA inference.

```
$ physeter.pl *.blastx --outfile=contam.report \
  --taxdir=gtdb-taxdump/ --taxon-list=phylum-taxa.idl \
  --tax-min-hits=2 --tax-max-hits=50 \
  --tax-score-mul=0.7 --tax-min-lca-freq=0.85
```

Other options can be applied to filter the BLASTX hits used for contamination assessment. Those are `--tax-min-ident`, `--tax-min-len` and `--tax-min-score`.

K-fold mode

The last functionality of `physeter.pl` is the *k-fold mode*. In this mode, the `DIAMOND` database is randomly split into 10 subsets. Then, `physeter.pl` runs 10 times and, for each run, hits from one of the subsets are ignored. The results of the 10 analyses are written in the standard output file. None of the Kraken-like file,

Anvio-like file, Krona-coompatible file and LCA file are available when running in *k-fold mode*.

```
$ physeter.pl *.blastx --outfile=contam.report \
  --taxdir=taxdump/ --taxon-list=phylum-taxa.idl \
  --tax-min-hits=2 --tax-max-hits=50 --k-fold=database.gca
```

The `database.gca` file is the list of all NCBI GCA/GCF accessions of the genomes used to build the `DIAMOND` database.

```
$ grep \> database.faa | cut -f1 -d'|' | cut \
  -c2- | sort -u > database.gca
$ head database.gca
```

```
1169474
1169539
1169540
1202447
1255295
127563
130081
1321669
13642
1389228
```

```
$ tail database.gca
```

```
GCF_900095815.1
GCF_900095855.1
GCF_900105895.1
GCF_900120375.1
GCF_900128725.1
GCF_900128965.1
GCF_900129645.1
GCF_900143135.1
GCF_900155405.1
GCF_900155645.1
```
